# Characterization of Group Behavior of Corruption in Construction Projects Based on Contagion Mechanism

**DOI:** 10.1155/2022/8456197

**Published:** 2022-03-19

**Authors:** Jingjing Li, Qiangqiang Shen, Wencan Gao

**Affiliations:** School of Traffic and Transportation Engineering, Changsha University of Science and Technology, Changsha 410114, China

## Abstract

With the rapid development of construction projects, more and more engineering corruption problems have emerged. Therefore, this paper proposes a SEIR (susceptible-exposed-infected-recovered) based corruption model to better understand the propagation process of corruption cases in construction projects. In this model, the data samples are collected from the 2018 Engineering Corruption Case Judgment Document, the propagation parameters are obtained through actual case analysis with the help of complex networks, the change process and key influencing factors of actual nodes in engineering corruption cases are simulated by Python. The study results indicate that the personnel conforms to the “4–9 transmission law,” in which the early stage is a period of high incidence of corruption cases. The network of corruption cases is somewhat vulnerable, and its spread is about minus 8 times the change in crackdown rate and 10 times the change in infection rate. The variation range of the susceptible population S and the removed person *R* in the propagation simulation curve can predict the relationship between corruption infection rate and crackdown rate, which can provide theoretical guidance for preventing the occurrence of corruption.

## 1. Introduction

Corruption is extremely harmful to social stability, may hinder economic development, and affect the development of political parties [1, 2]. Corruption in construction projects has become a key factor affecting the construction market environment and the sustainable development of external construction [3]. How to effectively punish and prevent corruption in the construction projects has become a challenge all over the world [4], especially in developing countries such as China [5–12], Ghana [13–15], Turkey [16], Brazil [17], and Iran [18]. Moreover, the construction industry is one of the “disaster areas” prone to corruption, which has seriously restricted the economic development [10]. Corruption cases in the construction projects may cause greater losses and more serious social impacts than general corruption cases because of some of its characteristics, such as multiple and complex participants, clear division of labour, hidden exchange of interests, serious corruption, long incubation period and difficult to play a supervisory role in the power supervision mechanism [19]. The huge investment in infrastructure during China's rapid urbanization can also lead to corruption in the construction projects [7, 20, 21]. In order to prevent corruption in the construction projects, the government has continuously improved regulations and increased the intensity of filing and reviewing cases of violations of laws and disciplines. Advanced screening through the official website of China Judicial Documents revealed that the number of first instance judgments on corruption cases in the engineering industry dropped from 5062 in 2016 to 1718 in 2020. Although the number of related cases has been controlled to a certain extent, the related problems are still very serious [2]. In conclusion, it is imperative to explore the propagation mechanism of corruption in construction projects for better preventing corruption in the construction projects.

Previous studies have analysed the characteristics of corruption in construction projects from behavioral and sociological perspectives, mainly through the data collection methods such as interviews, case studies, and questionnaires. Yu [22] found that the corruption of Chinese construction industry by using association rules is age-related, with the increase of corruption as managers approach retirement age. However, Zhang [23] pointed that the key factor affecting corruption in the real estate sector is not age growth, but abuse of power based on data from 135 cases. Emmanuel [19] summarized that the contract phase and the postcontract phase may be the main corruption prone stages of construction projects in many developing countries. It is notable that the professional backgrounds of corrupt elements are becoming more and more profound, the means of crime are diversifying, and the number of collective corruption is increasing [11, 23–26]. On the other hand, some scholars have analysed many cases to explore the criminal motives of corruption in the construction projects and the relationship of criminal personnel structure from the perspective of criminology. It is found that flawed legal systems, higher profits, and group complexity of construction projects are the key causes of corruption in construction projects [17]. Diviák [27] prompted that the criminal aggregation network of corruption presents a clear core-periphery structure in many cases, where all involved officials are located in the core of the network and have strong concealment in the corruption network. Wang [5] believed the corruption networks expand through brokerage activities in the form of spanning institutions and enlisting strategic resource holders. From the brief literature review, it can be found that many studies have explored the problem of corruption in construction projects from different perspectives, and illuminated the characteristics of corrupt behavior in construction projects such as complexity, dynamics, concealment, collective nature, and flexibility. It also verifies that individual characteristics have a strong correlation with the degree of corruption in both construction projects. However, these studies focused on the individual corruption characteristics from a behavioral or micro perspective, lacking group evolution context and macro insights. In addition, the research methods are still somewhat subjective such as questionnaires, expert interviews, literature reviews, which lack objectivity. Considering the dynamic nature of corruption behavior in construction projects, this study believes that it is also very important to explore the evolutionary relationship of corruption behavior propagation in construction projects from a macrogroup perspective.

To bridge the research gap, this study will explore the evolutionary process of the spread of corruption in construction projects by adopting the complex network theory and infectious disease SEIR model. This paper attempts to illuminate new ideas for preventing corruption in the construction industry from a macrogroup perspective. The parameters of the infectious disease model are obtained by actual corruption cases through the analysis of complex networks. The model can well describe the propagation mechanism of corruption behaviors in actual groups and is verified by actual corruption cases. The simulation results reveal the characteristics of corruption nesting in the engineering industry and the key factors of corruption governance, thus put forward governance recommendations for reference. Compared with the existing studies, the contributes of our research are highlighted as follows: (1) adopting a dynamic perspective to study the spread characteristics of corrupt behavior, (2) using a more objective method to determine the model parameters by extracting the actual case data through network feature parameters, and (3) improving the infectious disease model to prevent the corrupt behavior of engineering construction.

## 2. Literature Review

### 2.1. Characteristics of Corruption in Construction Projects

Corruption in the construction sector has spread to other industries and almost all the construction phases [28], especially the land grant stage, the bidding stage, and the construction stage [23]. In addition, the corruption cases in construction projects may cause greater losses and more serious social impact than the general ones. Furthermore, it is found that the types of construction projects corruption are determined by the conditioning variables such as type of leadership, position level, region, and age [22, 23, 29]. Among which, the age of 46–50 and 55–60 are the high incidence of corruption cases [22, 23], and engineering corruption is more likely to breed in second-and third-tier cities than in first-tier cities [22, 23]. These characteristics of corruption in construction projects have become a common focus of attention and seriously affected the healthy development of the engineering industry.

In order to further study the characteristics of corruption and reveal the causes of construction corruption, many scholars often explored the influencing factors of corruption by using questionnaire surveys [6, 7], expert interviews [4, 30], literature reviews [4, 31], and multiple case studies [16, 28, 32]. The causes of construction corruption are mainly psychosocial specific reasons, organization-specific reasons [31], regulations specific reasons, and project-specific reasons [31]. Firstly, a flawed legal system may provide opportunities for corruption in the construction industry [7, 10, 11]. Secondly, higher profits may be the another reason for corruption in the construction industry [30]. Thirdly, the complexity of the construction industry, such as the long construction period of the project and the complexity of the participants, makes corruption prone to occur [24, 31]. Finally, government officials play multiple roles in public construction projects, such as decision-maker, approver, project fund owner, and arranger, is easy to intervene the implementation of normal construction projects such as bidding activities [15]. The factors influencing corruption in construction projects are complex and variable, which also reflects the value of exploring the dynamic evolutionary relationship of the analysis of the law of corrupt behavior in construction projects.

The people who are in the core network will also change over time due to the dynamics, complexity, and concealment of the corruption network of the construction project [29, 33, 34]. Similarly, some scholars found that the network structure adjusts with time in the evolution of drug crime networks, and roles have great flexibility and variability at different mission stages and at different times [34–36]. Nekovee [37] quantitatively analysed the infection of corruption within the organization from a dynamic perspective, and found the lower threshold of corruption infiltration in the organization with a flat structure compared with the higher structured organization. Wang [5] found that the core of corrupt networks expanded by crossing institutions and securing strategic resource holders through brokering activities. Due to the versatility and flexibility of corrupt groups, corrupt groups spread through specific pathways within a certain spatial and temporal context [5], and such interactions against groups can be considered as the contagion of corrupt behavior [38]. Social contagion is similarly defined by scholars as “transmission among individuals in a group through interactions between individuals” [37, 38]. The current corruption phenomenon has shown a “fissile spread,” which spreads from individuals to groups, and even to the whole organization according to management logic. Similarly, the research on corruption behavior in construction projects still focused on the static or micro perspectives such as individual behavioral characteristics, corruption behavior influencing factors, corruption governance measures, and corruption degree assessment. In fact, the corrupt behaviors among corrupt groups in construction projects are contagious, and the interactive, dynamic and complex nature of the spread of these behaviors need to be taken into account. It is also very important to explore the evolutionary relationship of corruption behavior propagation in construction projects from a macrogroup perspective.

### 2.2. Theory of Complex Networks and Infectious Disease Models

Complex network is a theoretical tool for evaluating the operating status of complex systems that has been widely applied to various fields of construction management industry [27]. Generally, the various elements and their relationships within a complex system are abstracted into a network structure diagram. The traditional construction method is to transform each entity of the complex system into a network node, and transform the connection of entities into the connection of nodes [5, 27]. Graph theory is a useful tool to further study the complex network structure such as complex system evolution mechanism, diffusion mechanism and governance strategy and other scientific problems that has emerged in recent years [39].

Complex network of epidemic spread model on has attracted great attention among researchers of physics, mathematics, and epidemiology due to its success in predicting and controlling epidemic spread in reality [40, 41]. Fundamentally, the traditional spread of public opinion, epidemics and violations are simulated by SIR and SEIR [42]. The outbreak of COVID-19 has initiated a large number of numerical studies by using epidemiology models [43–46]. The propagations of diseases, behaviors and information in real systems are rarely independent of each other, but they are coevolving with strong interactions [40]. Fan [47] proposed a contagion model as a simple and powerful mathematical approach for predicting the spatial-temporal evolution of the onset and recession of floodwaters in urban road networks. Nekovee [37] studied the contagiousness of corrupt behavior within the organization, and its transmission behavior is somewhat similar to rumours or infectious disease. It is feasible to use infectious disease models to study the evolutionary pattern of the spread of corrupt behavior.

A systematic review of the literature reveals that corruption in construction projects is a hot area of research for scholars nowadays. Due to the concealment, complexity and seriousness of corruption in construction projects, the management of corruption in construction projects is also the common goal of all countries nowadays. The dynamic nature of corruption networks is illustrated by the close division of labour and the mutual change of roles among the personnel of corrupt groups. At the same time, the infiltration of internal corrupt practices illustrates the contagious nature of corrupt practices. Intermediaries expanded the core of the corruption network, and transmitted the interactions between groups to jointly constitute the complex network of corrupt practices. In addition, infectious disease dynamics model with complex networks have proven to be a key approach for the study of behavioral transmission and have been widely used in various fields. The systematic discussion also provides a rich theoretical foundation and guiding direction for this study. However, the current research on behavioral communication mainly focused on the field of public opinion governance and infectious disease surveillance, and lacked the behavioral communication of corrupt groups in the construction projects field. Most scholars just concentrated on the individual characteristics and construction project corruption features from a behavioral perspective. Therefore, the spread characteristics analysis of construction corruption in this study is a new exploration of the regulation and governance of the construction management industry. Dynamic research on the changing characteristics of different periods is also the key point of this study. To address the research gap, this paper adopts the complex network theory and the SEIR model of infectious disease to study the propagation process pattern and governance measures of corruption cases in construction projects from a dynamic perspective.

## 3. Construction of an Infectious Disease Model for Corruption Nesting in Construction Projects

Infectious disease behavior on complex networks has been widely used by scholars in various fields. The method of infectious disease dynamics is a mathematical technique that has been developed into a rich interdisciplinary field. Scholars found that the transmission mechanism of infectious diseases is similar to information or human behavior [42]. Infectious disease models have been widely used in various fields such as the prediction and simulation of the new coronavirus [43, 48–50], the spread and intervention of unsafe behaviors of construction workers, flight operation risk spread analysis, panic spread research, the spatial spread and temporal evolution of the onset and recession of floodwaters in urban road networks [42, 47, 51, 52]. Besides, infectious disease models are used in the study of behavioral communication, which can reflect the propagation and evolution process of behavior at the microscopic level, and predict the trend of behavioral diffusion at the macroscopic level [53].

Corruption in construction projects has the characteristics of dynamic and contagious, and its evolutionary pattern can be analysed by infectious disease models. The emergence, replication, and spread of corruption in the engineering field are very similar to the transmission process of infectious diseases [42]. The spread of infectious diseases in the population requires the source of infection, latent persons, susceptible groups, and good conditions for transmission. Generally, the corruption nest of a construction project can be regarded as a transmission process from single individual corruption infection to group corruption infection. Considering that there are generally many people involved in the corruption case of a construction project, and the exchange of benefits is concealed, there may be an incubation period for individual corruption [19]. In addition, the corruption of construction projects has a certain latent nature because some corrupt groups or individuals would weigh the pros and cons before deciding to participate in corrupt practices. The traditional SIR model of infectious diseases does not consider the incubation period of corruption [54]. The spread study of corruption cases is based on the traditional SEIR model [55], which assumes that the overall propagation process of corruption nesters in construction projects conforms to a single process within a system from a state of vulnerability to corruption to participation in corruption and finally to being cracked down and caught. Therefore, this paper adopts an improved SEIR model and adds the incubation period parameter [47] to more reasonably explore the transmission mechanism of corruption behaviors in construction.

**Figure 1:**
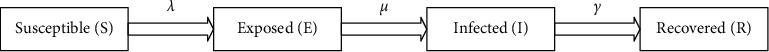
State transition diagram of the SEIR model.

In formal rumor-spreading models, a closed population is subdivided into three groups; those who are ignorant of the rumor, those who have heard and actively spread it, and those who have heard but ceased to spread it [37, 42]. This paper designs a SEIR model for the propagation of corruption nests in construction projects with four populations, including susceptible population (*S*), exposed population (*E*), infectious population (*I*), and recovery population (*R*) [55]. The propagation state of the model is shown in Figure 1. Moreover, the evolutionary relationship of corruption transmission in this study is revealed by the SEIR model that integrated various external real factors to obtain the change rate of infectious diseases.

In this paper, it is assumed that the total number of nodes in the project corruption network is *N* and the number does not change throughout the propagation process, and the information propagation time is represented by *t* [50]. This study hypothesizes that corrupt behavior is generated through intergroup organizational interactions. The information propagation process in the SEIR model is described as follows.

1. The susceptible population refers to the group who are prone to participate in the illegal and corrupt behavior of the construction projects.
2. The latent population refers to the group who have been exposed to the corruption behaviors but have not yet decided to participate and has no transmission motivation.
3. The people infected by corruption refers to the group who participate in the case of corruption or commit corruption and have the motivation to spread corruption.
4. The dismissed people refer to the group that have been cracked down by external discipline inspection departments, courts, and other channels and are in a state of being dismissed.
5. When *t* = 0, corruption of the initial project generated in the network. The susceptible nodes in the network first convert to exposed nodes with the probability of *λ*, and then the exposed nodes convert to the infected node with the Probability of *u*. Finally, the infected nodes can be converted into the Remove nodes with the probability of *r*[44].
6. The overall process of spreading corruption in engineering projects can be expressed as follows: if the susceptible state individual *m* is considered to have corruption behavior with neighbouring corrupt individual *j* in the period [*t*, *t* + 1], then susceptible state individual *m* will be in latent state *E* by deciding whether to participate in corruption or not. When individual *m* in the latent state participates in corruption by choice, individual *m* will become a member of the corrupt group I in the construction projects. When individuals *m* in a corrupt state are under the supervision of external government departments and other relevant agencies, they are eventually arrested and become a removal group *R* [42]. In the whole process of corrupt spreading behavior in construction projects, each node may involve in corruption, which mainly depends on the comprehensive impacts of multiple factors.
7. During the initial propagation of corruption in construction projects, it is assumed that the people in the group will be in one of the states where corrupt interactions occur with their neighbouring nodes during the beginning of corruption propagation.

The dynamic differential equation expression of the infectious disease model is shown in equation.

(1) $$\begin{array}{c} \left\{\begin{array}{c} \frac{\text{d}S\left(t\right)}{\text{d}t}=-\lambda *S\left(t\right)*I\left(t\right),\\ \frac{\text{d}E\left(t\right)}{\text{d}t}=\lambda *S\left(t\right)*I\left(t\right)-\mu *E\left(t\right),\\ \frac{\text{d}I\left(t\right)}{\text{d}t}=\mu *E\left(t\right)-\gamma *I\left(t\right),\\ \frac{\text{d}R\left(t\right)}{\text{d}t}=\gamma *I\left(t\right),\\ N=S+E+I+R. \end{array}\right). \end{array}$$

Among them, *S* (*t*) represents the density of vulnerable nodes, *E* (*t*) represents the density of latent nodes, *I* (*t*) represents the density of infected nodes, *R* (*t*) represents the density of attack removal nodes, *S* (*t*) + *E* (*t*) + *I* (*t*) + *R* (*t*) = 1. *λ*, *μ*, *γ* represent the probability of conversion between personnel at each node, 0 ≤ *λ*, *μ*, *γ* ≤ 1, *λ* is the corruption infection rate, *μ* is the potential decision rate, and *γ* is the crackdown rate.

The infection rate *λ* refers to the probability that an infectious disease will infect from a susceptible population to an individual in a complex network, mainly refers to the probability that a susceptible population becomes a potential individual node in the corrupt network. The incubation state refers to the incubation period of the infectious disease from the individual infection state to the outbreak stage of the group infection. In the corruption case network, the potential decision rate *μ* is used to express the probability that the potential individual corrupt personnel is willing to participate in the corruption case after the balance of income and expenditure analysis. The crackdown crate *γ* refers to the probability that the infected node is cured by some measures, which also means the probability that the official organization arrest the corrupt personnel.

## 4. Model Parameters

The parameter of the infectious disease model generally is a measurable indicator. Previous studies set the relevant parameters mainly through historical data, related literature, genetic algorithms and particle swarm optimization algorithms [42, 45, 46, 48, 56].

### 4.1. Data Collection

There are many news and network reports on corruption cases of construction projects, but there are two problems: description bias and selection bias [27]. Given the sensitiveness and implications of criminal proceedings, criminal intelligence and investigations strive for achieving the most accurate representation of each case [57]. China Judgments Online is an official platform sponsored by the Supreme Court [20], which provides numerous corruption cases including the construction projects. Moreover, the China Judgment Online records in detail the interaction of corrupt persons and other groups in the construction projects. In order to ensure the validity and authenticity, the data in this paper are derived from the legal judgment documents published in China Judgments Online.

This paper uses the advanced filtering function of the China Judicial Documents to obtain all the data. In the process of data screening, this study sets the case type, the trial procedure, court level, year, and document type as “criminal case,” “criminal first instance,” “all,” “2018,” and “judgment” respectively, and then conducts a full-text screening for “bribery,” “acceptance of bribes,” “construction projects” and “project corruption.” After screening, analysing and sorting out specific content, a total of 2158 engineering corruption cases were found, involving people in 12 provinces and 1 municipality.

The cases of corruption in construction projects were filtered according to the following criteria: (1) The information of all judgment documents is complete, and the content of which is related to the field of construction projects. (2) All the judgment documents should be read in detail and adequately hand-screened to find interrelated cases. Based on the defined filtering rules. Then, a total of 109 cases with the most complex case relationship, large amount of corruption and long latency are selected for analysis. The cases selected in this paper are suspected of several charges, including bribery, acceptance of bribes, abuse of authority, collusion in bidding. All actors named in the selected adjudicative instruments as being involved in illegal activities are included in the detailed detail record. The earliest of the arrested core criminal officers began participating in corrupt activities in construction projects in November 2007 and were arrested and prosecuted in late 2017. Analysis of the data shows that nodes 2, 4, 5 and 34 are government department officials. The rest of the nodes are contractors except for the group of people associated with government officials. In addition, nodes 1, 2, 3, 4, and 5 are the key individuals who were eventually apprehended for participating in or committing corrupt acts on the construction projects. The selected cases involved 34 persons and institutions, more than 14 million single-person crime, and an incubation period of up to 10 years. The five adjudication documents of the selected cases are all first instance judgments of criminal cases, numbered (2018) Xiang 1224 No. 17, (2018) Xiang 1224 No. 64, (2018) Xiang 1224 No. 65, (2018) Xiang 1224 No. 196, (2018) Xiang 1281 No. 175 respectively.

In this paper, all names of personnel and company are replaced by numbers for conveniently analysing the relationship between corrupt personnel, then a 34 *∗* 34 one-dimensional adjacency matrix and a directed engineering corruption nest personnel relationship network are constructed to determine the parameters of the infectious disease model.

### 4.2. Partial Model Parameter Settings

This study determines the relevant parameters through the analysis of corruption cases in actual construction projects. Corruption contagion in actual construction projects can be initiated by several people or a small group; whereas the corruption spread of the construction projects involved in this case is initiated by one person and then gradually expands based on the detailed analysis of the five legal judgments involved in this study. Therefore, this paper selects one key person as the source group of corruption communication. The total number of people in the network is assumed to be *N* = 34 by analysing the actual case. *S*, *E*, *I*, and *R* represent the initial number of personnel at each state node. Therefore, this study assumed the number of each group as *I* = 1, *E* = 0, *R* = 0, and *S* = 33 in the initial state, respectively. Due to the complexity, dynamics, concealment and sensitivity of corruption cases in construction projects, it is difficult to directly quantify the management measures such as supervision, close visits and talks in corruption cases in construction projects. For the setting of the crackdown rate of corruption in construction projects this study is replaced by the number of corrupt group members arrested and imprisoned. Considering that five people have been arrested in the organization's 34-person corruption network, the rate of attack is *r*=5/34=0.147; The potential decision rate *μ* depends on the cost-benefit risk of corruption involved in the project, with a random possibility each time; The potential corruption decision rate *u* of corrupt personnel is taken as 0.5, in order to make this group of personnel have the same possibility each time. The analysis found that the incubation years of the five corruption offenders were 10 years, 9 years, 7 years, 5 years, and 4 years, respectively. The propagation time of 10 years in this study is the maximum incubation time for all groups of people and institutions involved in construction corruption projects. Therefore, this paper will use the incubation time as the parameter of the propagation time to describe the interaction of the group's corruption behaviors during the entire propagation period.

### 4.3. Corruption Infection Rate Parameter Setting

This study attempts to determine the magnitude of personnel corruption contagion rate in the construction projects through complex network modelling. At the node level, degree centrality is used to represent the structural position of actors in a network [58]. The nodes with greater degree centrality in complex networks can be regarded as important conduction nodes [29, 58, 59]. In this study, the higher the degree of node centrality in the corruption network of the construction project, the more people have corrupt behavior. Therefore, this study reveals the number of corruption contagions in the corruption nesting network of construction projects according to the connection effect of several important nodes with greater centrality [29]. The corruption infection rate is defined as the average contact degree of the core personnel, as shown in equation.

(2) $$\begin{array}{c} \lambda =\frac{1}{n}\sum_{i=1}^{n}C_{i}. \end{array}$$

Among them, *λ* is the corruption infection rate, *C*_*i*_ is the node ratio of the *i*-th core corrupt personnel to the corruption network, *i* is the *i*-th core corrupt personnel in the corruption network, and *n* represents the number of core personnel in the corrupt network.

#### 4.3.1. Complex Network Model Construction

**Figure 2:**
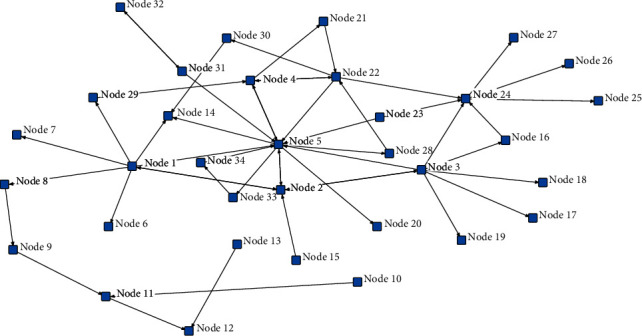
Personnel contacts of construction corruption case under complex network.

Considering the complexity and interaction of corrupt behaviors among corrupt groups in construction projects, it is difficult to visualize the connections between nodes through textual narratives, and the use of complex networks can be a good way to visualize the relationships among nodes. Based on the five selected adjudication documents, the relationship between groups of people is analysed in detail. If there is corruption between any two individuals in the corrupt group, a link is established. The corrupt personnel and the corruption relationship between personnel are abstracted as the network nodes, the edge of the network respectively, then the corruption relationship network is established [5, 60]. The corruption network can be represented by the adjacency matrix *An* *∗* *n*, so that if there have been corrupt behaviors between personnel, then *a*_*ij*_ = 1, otherwise *a*_*ij*_ = 0. The adjacency matrix can reflect the relationship between groups in the complex network, as shown in Table 1. Ucinet software can build a corruption network for the corruption case group of construction projects based on the relationship of the adjacency matrix. A complex network of the personnel contact in the engineering corruption case is built by using Ucinet software, as shown in Figure 2. A detailed adjacency relationship matrix data is shown in the supplementary materials Annex 1.

#### 4.3.2. Corruption Infection Rate in Complex Networks

The nodes with larger out-degree and in-degree values can be considered as the important conduction nodes of the network in this study. In addition, it can be considered that the formation of corruption network is due to the corruption of a group or multiple individuals. Betweenness centrality is used to measure the transportation capacity of nodes in a complex network. The higher the betweenness centrality, the more influence the node has. It is believed that the nodes with high intermediary centrality in the corruption network of construction projects have strong corruption transmission capabilities. Closeness centrality is a characteristic value that reflects the distance between a node and the centre of the network [27, 29]. Thus, it is significant to calculate the degree centrality, betweenness centrality, closeness centrality, and core edge analysis of the corruption case network, so as to determine the key personnel in the corruption network [27, 39]. This study assumes that the corruption contagion rate of the corruption case is determined by the average degree centrality of the core personnel. The centrality indicators of each corrupt network node are shown in Table 2. Detailed data can be found in supplementary materials Annex 2.

Table 2 reveals that the top eight nodes with the highest total degree of centrality are: 5, 1, 3, 2, 22, 24, 4, and 11 respectively. The order of nodes with high betweenness centrality is 5, 2, 1, 22, 3, 4, 24, 8; The distribution of nodes with more tightness centrality is 2, 3, 1, 31, 5, 22, 4, 34. According to the degree of close connection between nodes in the complex network of the corruption case, the core and edge personnel in the corruption network are determined by using Ucinet software [35, 57, 59], as shown in Table 3.

According to the three-centrality analysis and core-edge structure of the corruption network personnel in Table 2 and Table 3, 7 people, namely Node 1, 2, 3, 4, 5, 22, and 24, are identified as the core personnel of the corruption network in this case. The corruption contagion rate of the project is *λ*=0.239 according to the above assumption of equation (2). In summary, this paper uses the network characteristics of the case personnel to determine the improved SEIR model parameters, as shown in Table 4.

## 5. Simulation Verification and Analysis

### 5.1. Evolutionary Relationship of Corruption Contagion in Construction Projects under Actual Cases

**Figure 3:**
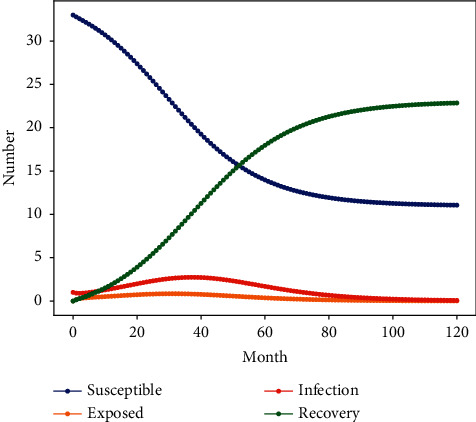
Variation in the nodes number of personnel during corruption with actual parameters.

This paper defines the total number of research groups as *N* = *S* + *E* + *I* + *R*. Where *S*, *E*, *I* and *R* represent the number of personnel at each status node in the process of corruption propagation. The corruption infection rate *λ* of initial corruption cases is 0.239. Potential decision rate *μ*, crackdown rate of the formal organization *γ*, and corruption propagation time *t* are 0.5, 0.147 and 10 years respectively. This paper use Python to simulate the infectious disease model in the state of corruption nest, then obtain the evolution results of people in each stage of the spread process of corruption nest, as shown in Figure 3.

Figure 3 shows that the number of corrupt personnel I node will first increase to a certain level and gradually decrease over time, then stabilize, and finally reach a peak in three years. The number of individuals at the latent node *E* is consistent with the changing trend of corrupt personnel as a whole, and the peak value is less than the number of corrupt personnel. It demonstrates that in a relatively closed network structure, the construction project-related personnel who would like to participate in corruption will eventually become a member of corruption to externally intervene in the construction project. The number of groups *S* in the perishable stage declines in a power law over time, indicating that the degree of corruption is relatively serious.

The number of *R* in an immune-strike state shows an increase in the power rate, which shows a good effect in combating corruption by the official organization. Throughout the propagation, the crackdown by official organizations and the spread of corruption coexisted, intersecting in about 4 years. Furthermore, the simulation curve of corruption propagation reveals that the effect of corruption cracking is significantly stronger than the level of corruption after 4 years, and the number of people in each state cluster is basically in a stable state after a latent period of about 9 years, which is consistent with corruption incubation period of around 10 years in the current construction project [23]. On the one hand, it illustrates the significant effect of the crackdown by official institutions in reducing corrupt practices during their dissemination online. On the other hand, it also shows that the complex and closed corruption network structure will eventually collapse by itself after a certain time of spreading due to the sparseness of the network and the multiple complexity of the personnel. This is also evidence of the vulnerability and poor flexibility of corruption case networks in long-term propagation.

The source data of five selected engineering corruption cases reflects that the time of 80% corruption criminal to form a group of corruption is less than 4 years. It also illustrates that the corruption criminal will quickly form a network of corruption in various ways under the premise of satisfying the profit of all parties. In addition, it can also be found that the parameter settings are based on the actual construction project corruption cases in this study. The peak number of personnel for corruption propagation throughout the construction project is the same as the number of core personnel for corruption apprehension in the actual case. At the same time, by observing the evolution of the corruption propagation pattern, we can find that only five of the most central corrupt people in a corrupt network of 34 people were arrested, which is also consistent with our actual data of corruption cases in the magisterial documents. Other groups of people involved in corruption in construction projects may have been prevented from corruption by a variety of measures such as external supervision, confidential visits and interviews, combat and arrest only the most central key personnel, which is similar to the realistic measures to prevent corruption in construction projects.

The results show that the corruption and infectious disease model constructed in this paper is feasible and consistent with the actual situation. The “4–9 propagation law” of construction project corruption nest cases was discovered through actual case analysis.

### 5.2. The Impact of the Formal Organization's Crackdown Rate on the Spread of Corruption

**Figure 4:**
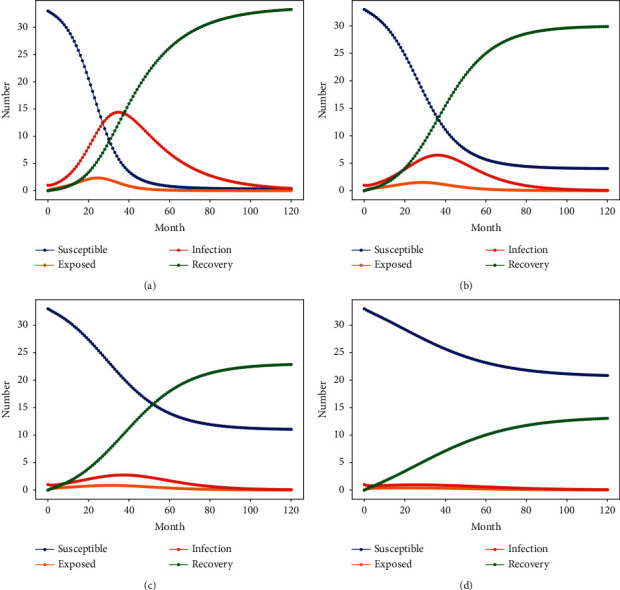
The relationship of changes under different crackdown rates. (a) *γ* = 0.05, (b) *γ* = 0.1, (c) *γ* = 0.147 (d)*γ* = 0.2.

This study sets the parameter of crackdown rate and others based on the specific construction projects corruption cases to investigate the effect of the crackdown rate on the contagion behavior in the construction project. Under the condition of constant corruption infection rate of 0.239 and other parameters, the crackdown rate *γ* of the formal organization is set to be [0, 1]. Variable parameters should be allowed to vary within a certain range to better reflect the real situations of corruption contagion behavior. However, when the parameters of corruption transmission behavior are freely changing, the simulation of corruption transmission behavior for construction projects does not truly reflect the correspondence under the changes of corresponding factors. More importantly, the factors under the infectious disease model are fixed parameter variation studies under transmission behavior analysis [42, 46, 48]. To overcome these shortcomings, this study uses sensitivity analysis to explore corruption contagion behavior under several relatively varying scenarios. Taking into account the actual crackdown rate of 0.147, the parameter values of the crackdown rate of the formal organization were set to 0.05, 0.1, 0.147, and 0.2, respectively, by sensitivity analysis of the crackdown rate [42, 61]. Based on the infectious disease model, Python is used to simulate the crackdown rate of personnel informal organizations. The change in the number of personnel at each node in the state of corruption is shown in Figure 4.

Figure 4 and Table 5 show that when the crackdown rate of formal organizations exceeds 0.2, corruption will hardly occur. With the increase of the crackdown rate, the peak value of corrupt personnel nodes I gradually decreases and the propagation time to reach the peak value gradually increases. When the corruption crackdown rate is increased from 0.05 to 0.1 by 5%, the degree of corruption is relatively reduced by 42.85%, and the peak propagation time of corruption is relatively increased by 8.57%. When the corruption crackdown rate increased by 9.7% from 0.05 to 0.147, the degree of corruption decreased by 78.57%, and the propagation time of corruption at its peak increased by 14.29%. It can be seen that the crackdown rate is negatively correlated with the degree of corruption in construction projects, and positively correlated with the peak propagation time of corruption. The corruption spread, the propagation time of corruption cases reaching the peak is about minus 8 times, 1.6 times of the crackdown rate respectively.

The number of perishable personnel *S* and the number of immune-strike personnel *R* also show power-law changes, and the magnitude of the change in the two curves reflects the relationship between corruption and crackdown in the corruption case. It can be seen from Figure 4 that when the crackdown rate is 0.05, 0.1, and 0.147, the susceptible *S* and the remover *R* intersect at 2.5 years, 3.2 years, and 4 years, respectively. The size of the intersection reflects the speed of the spread of personnel corruption in the corruption nest case. The whole corruption nest case network is in a stable state. When the crackdown rate increases by 5% and 9.7%, the increase of *s* of corrupt personnel in the project is 150% and 500%, respectively, which is 9.375% and 31.25% greater than the decrease of *R* of corrupt personnel in the project. With the increase of the crackdown rate, the increase of perishable personnel *S* in the construction project is greater than the decreased number of personnel removed from the immunization crackdown of the official organization of the construction project. It shows that the number of people involved in the spread of corruption is far smaller than the effect of the corruption crackdown, and the crackdown rate of the corruption case of the project is also relatively high, which meets the crackdown requirements.

The analysis shows that the occurrence of corruption prevention cases can be reduced from the source through the dynamic monitoring of the project and strengthening the crackdown on the organization.

### 5.3. The Impact of the Infection Rate of Corrupt Personnel in the Engineering Corruption Case on the Spread of Corruption

**Figure 5:**
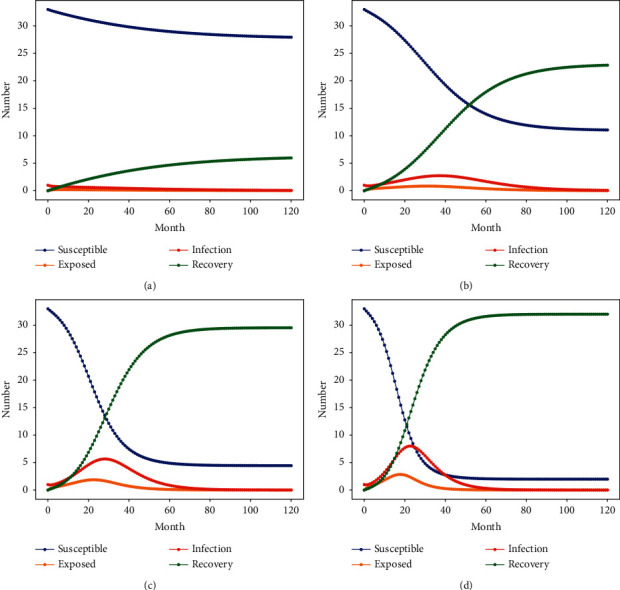
Relationship between changes under different infection rates. (a) *λ* = 0.139, (b) *λ* = 0.239, (c) *λ* = 0.339, and (d) *λ* = 0.439.

Under the constant condition of crackdown rate (i.e., 0.147) and other parameters, the value of the corruption infection rate *λ* of the personnel in the engineering corruption network is [0, 1]. Considering the actual corruption transmission rate of 0.139, the values of corruption transmission rate parameters were set to be 0.139, 0.239, 0.339, and 0.439 by sensitivity analysis of the transmission rate [61]. Based on the infectious disease model, the extent of corruption nesting in construction projects under different corruption contagion rates is simulated by Python. The personnel change of each node in the state of corruption is shown in Figure 5.

According to the simulation results in Figure 5 and Table 6, it can be seen that corruption hardly occurs when the corruption infection rate of a construction project is less than the crackdown rate. When the corruption infection rate increases by 10% from 0.239 to 0.339, the transmission time of corruption nest cases reaching the peak decreases by about 25%, and the peak number of people involved in corruption increases by about 100%. When the corruption infection rate increases by 20% from 0.239 to 0.439, the propagation time of corruption cases reaching the peak is relatively reduced by about 50%, and the peak number of people involved in corruption is relatively increased by about 200%. The transmission scale and rate of corruption network increased with the increase of the change range of corruption infection rate. The infection rate is positively correlated (i.e., 0.1 times) with the corruption degree of the construction project, and negatively correlated (minus 0.4 times) with the time of the construction corruption reaching the peak of transmission. The infection rate of engineering project corruption has a great impact on the spread of corruption cases. The control of the infection rate of corruption must start from multiple aspects including real-time dynamic monitoring, reduce the spread of corruption, and ensure the normal implementation of the entire process of the project from the source.

In the whole process of corruption propagation, *S* curve and *R* curve show a power-law trend. Figure 5 shows that the simulation curves of the *S* curve and the *R* curve intersect at 4 years, 2.5 years, and 2 years, with intersection points of 6 people, 14 people, and 12 people respectively, as the infection rate increases. There will more perishable groups S and removed groups *R* over time. In a stable state of transmission, the relative reduction of personnel prone to corruption in the project is about 50% when the infection rate of corruption increases by 10%, which is greater than the increase of 27.27% by the formal organization of the project. The rate of decrease of perishable personnel in engineering projects is about 83.33% when the infection rate of corruption increases by 20%, which is greater than the rate of 45.45% in the official organization of the project to crack down and remove the increased personnel. It can be seen that the spread of the number of people involved in corruption infection is far greater than the effect of corruption attacks with the increase in the infection rate. In addition, the impact of the intensity of corruption infection rate will be greater when the reduction of corruption prone personnel is greater than the increase personnel in the formal organization of the project.

### 5.4. The Impact of Changes in Latency Period on the Level of Corruption in Construction Projects

**Figure 6:**
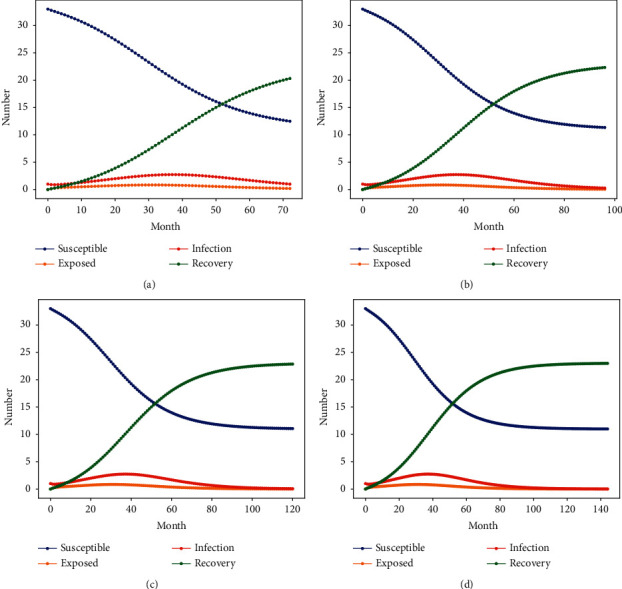
Relationship between changes under different latency periods. (a) *t* = 6 years, (b) *t* = 8 years, (c) *t* = 10 years, and (d) *t* = 12 years.

Parameters obtained from actual cases are taken as the corruption contagion rate and combating rate of construction projects. The influence of the evolutionary pattern of corruption nesting behaviors in construction projects under different incubation periods is studied separately. This paper selects the incubation period of 6 years, 8 years, 10 years and 12 years as the characteristic parameters to analyse the evolution relationship between the incubation period and the corruption degree of the project. Based on the infectious disease model, the extent of corruption nesting in construction projects under different corruption Latency Period are simulated by Python. The change of the personnel number at each node in the state of corruption is shown in Figure 6.

According to the simulation results in Figure 6 and Table 7, a very interesting phenomenon of corruption group evolution can be found when the rate of corruption contagion and the fight against corruption in construction projects are determined. The change of latency time has little effect on the peak value of group I in the construction projects corruption. When the incubation time is less than 10 years, the number of corruption-prone groups *S* and the number of removal-strike stage groups *R* are still in the process of propagation evolution. This also verifies the accuracy of the 10-year latency time for the five construction projects corruption adjudication documents selected in this study.

This is contrary to the traditional latency period of engineering projects and significant relationship between the degree of corruption. This is mainly because the number of corrupt groups in construction projects is relatively fixed in this study, except the relatively fixed values of the contagion rate and strike rate parameters that affect the behavior of corruption transmission in construction projects. Similarly, this evolutionary relationship illustrates that the length of latency time cannot influence the peak state of corruption contagion behavior in construction projects when the propagation parameters are in a determined state, unless the latency time is short enough to influence the formation of corruption nesting networks in construction projects. This also validates the infectious behavior of the corruption nest in construction projects will be rapidly infected to form a complex and hidden corruption network among the group in about 4 years.

This study demonstrates the behavior of corruption transmission in construction projects by changing the incubation time, and the simulation results also verify the rationality and scientific validity of the infectious disease model designed in this study.

## 6. Discussion

### 6.1. Findings

The construction and practical validation analysis of the construction projects corruption nesting SEIR model shows that the model constructed in this paper is scientific and reasonable in line with the actual situation. Due to the dynamics, complexity, and concealment of the corruption network of the construction projects, the people who are in the core network will also vary over time [27, 34, 36]. Zhang [42] proposed the MI-SEIR model considering the influence of media and interpersonal relationships on opinion dissemination based on the SEIR model. Nekovee [37] explored the mechanism of penetrating and spreading corrupt behavior within the organizations, and the behavioral interaction between corrupt group. The traditional spread of public opinion, epidemics and torts are generally based on models such as SIR and SEIR [42, 45]. The dynamic time dimension is used to study the propagation law of corruption in engineering projects according to the changing relationship of incubation time.

Because of the time lag between the occurrence of corruption and its apprehension, the length of the incubation period of corruption largely influences the extent of the spread of corruption in construction projects [19, 22, 23]. In the propagation model constructed in this paper, the relevant parameters are obtained from the actual case. The simulation results found that the level of corruption fight in a corruption network after a latency period of more than 4 years will be significantly stronger than the level of corruption propagation, and hardly exist after 9 years. Yu [22] found that the average latency period was significantly longer (i.e., 6.4 years) in construction-related corruption. There are 11 cases over 10 years, with the longest being 15 years. Zhang [23] also analysed the average corruption latency period of 8.02 years through actual data, which is also consistent with the nonexist corruption of about 9 years' latency time in this study. This shows that the corruption nest group in construction projects can quickly form a complex and flexible corruption network within the first 4 years. When the incubation period exceeds nine years, the corruption network of the construction project basically disintegrates and the core group in the corruption network will be hit.

The single factor analysis of corruption communication reveals that the corruption will hardly occur when the crackdown rate of formal organizations exceeds 0.2 or the rate of corruption infection is less than the crackdown rate, which shows that the corruption case network has certain fragility. There is a strong correlation between the crackdown rate, infection rate and the degree of corruption. Corrupt groups spread through specific pathways within a certain spatial and temporal context due to the versatility and flexibility, and such interactions against groups can be considered as the contagion of corrupt behavior [5, 38]. The increase of the crack-down inhibits the maximum scale and rate of corruption risks in the network, and the increase in the rate of infection determines the scale and rate of spread of the corruption case network. The spread scale of corruption cases, the propagation time reaching the peak is about minus 8 times, 1.6 times of the crackdown rate respectively. The spread scale of corruption cases is about 10 times of the corruption rate, and the spreading time is about minus 2.5 times of the infection rate. The best way to reduce the incidence of corruption cases is to prevent corruption infection and strengthen the crackdown. The serious phenomenon of corruption spreads from individuals to groups and even to the whole organization, and from lower levels to higher levels according to management logic.

### 6.2. Theoretical Implications

On the one hand, we try to explain and discover the corrupt behavior of construction projects and its laws by using the contagion theory model. It enriches the theory and method of construction project corruption behavior research, while expanding the application scope of contagion model theory. This paper analyzes the evolution relationship of corrupt behavior in engineering projects, and focuses on the evolution law of corrupt behavior groups. This study broadens the research field of corruption behavior in construction projects, enriches the scope and conditions of application of the contagious disease model theory to the contagious behavior of corruption in construction projects, and applies the contagious disease SEIR model to the contagious behavior of corruption in construction projects. By analysing the evolutionary relationship of corruption contagion behavior in engineering projects, the paper focuses on the changing relationship of corruption contagion behavior under the influence of factors such as external corruption contagion behavior and crackdown under the change of latency time. The study breaks the traditional research for the degree of corruption in construction projects, the characteristics of corruption, corruption governance under a single perspective to consider the problem starting. The nature of corruption contagion behavior is analysed and sorted out, which helps to understand the inner evolution law of corruption contagion behavior in construction projects and expand the boundary area of corruption research in construction projects.

On the other hand, the complex network theory used in this paper combined with the contagion model theory explores the characteristics of the spread of corrupt behavior from a dynamic perspective. The method of determining model parameters through complex networks obtains more objective data than the questionnaires and expert interviews used in traditional studies. It also provides a theoretical reference for scholars to adopt new methods for data acquisition and consolidates the exploration of scientific governance in the field of corruption management of construction projects. At the same time, the evolutionary relationship of corruption contagion behavior in construction projects also breaks the tradition of analysing the relationship characteristics of corruption behavior mostly from a static perspective. It also reveals the changing characteristics of group behavior under the whole time of corruption transmission, and also provides a new theoretical exploration for the targeted fight against the occurrence of corruption.

### 6.3. Practical Implications

The study provides clearer target and compelling rationale for policy makers to select the right strategic tool in their fight. First of all, in view of the prevalence of such corruption in construction projects, the fight against corrupt groups should use a variety of means for full process control. Secondly, considering the simulation analysis of the actual case dissemination process in this study, the early stage is a high-frequency stage prone to corruption in construction projects. Therefore, the government supervision department should attach great importance to the control of small groups in violation of regulations in the early stage of the project such as the bidding process. Thirdly, the corruption hardly occurs when the strike rate of formal organizations exceeds 0.2 or the corruption infection rate is lower than the crackdown rate. Therefore, the government supervision department can choose appropriate crackdown and supervision strategies to improve the efficiency of project management according to the degree of corruption of local construction projects. Finally, government departments should pay more attention to the informal organization groups in the construction process of construction projects, and strengthen the control of the generation and dissemination of inter-group violations.

## 7. Conclusions and Future Study

### 7.1. Conclusions

This study proposes an improved SEIR model of corruption contagion behavior in construction projects based on the epidemic model considering the variation of latency time of group personnel. Five adjudication documents collected from the official website of Chinese adjudication documents were used to validate the propagation model test of corruption nesting cases in construction projects. This paper demonstrates that the model is consistent with the process of corruption propagation in construction projects, explores the evolutionary relationship of corruption propagation, and investigates the effects of changes in crackdown rate and contagion rate on the contagion time and the degree of corruption. Based on the simulation results, the following conclusions can be drawn.

(1) The improved SEIR model for infectious diseases proposed in this paper is scientifically and rationally compatible with the actual process. (2) A complex and hidden network of corruption in construction projects can be formed rapidly within four years or even less. When the latent period exceeds 9 years, the corruption network of construction projects will basically disintegrate and the core group in the corruption network will be crackdown. (3) If can target to Enhancement of the crackdown rate or even some regulatory measures by external regulatory agencies can effectively reduce the spread and proliferation of corruption behavior at the early stage of corruption contagion behavior in construction projects. Otherwise, if external regulators allow corruption to occur, corruption in construction projects will rapidly spread throughout the corrupt community. (4) There is a strong correlation between the crackdown rate, infection rate and the degree of corruption, the increase of the crack-down inhibits the maximum scale and rate of corruption risks in the network, and the increase of the infection rate determines the scale and rate of spread of the corruption case network. The spread scale of corruption cases, the propagation time reaching the peak is about minus 8 times, 1.6 times of the crackdown rate respectively. The spread scale of corruption cases is about 10 times of the corruption rate, and the spreading time is about minus 2.5 times of the infection rate.

### 7.2. Limitation and Future Research Directions

Due to the limitation of the data source of corruption cases in construction projects, the dissemination process of corrupt behaviors in this study does not consider realistic issues from external factors such as investment in construction projects, local economic conditions, and government transparency. In addition, this study considers the parameter settings under real cases. However, the effect of free variation of factors in the interval is ignored because the parameters under the SEIR model of infectious diseases need to be fixed when analysing the effect of changing states of factors on the behavior of corruption transmission in construction projects.

The modelling analysis and feature disclosure in this article are based on a specific engineering corruption case; however, a complex network can be constructed with the help of big data techniques to determine the parameters of the infectious disease model.

In the context of Industry 4.0, the vigorous development of digital intelligence technology provides an efficient tool for the engineering management industry. In future research, the SEIR model of infectious diseases should be further improved when considering more influencing factors, such as the investment situation of construction projects, regional GDP, legal regulatory system, and local integrity index. The combination of digital intelligence technology and infectious disease models can dynamically predict the possibility of each behavior in real time. On the other hand, future research on corruption nests in engineering projects will consider multicase data to deeply analyse the evolutionary relationship of propagation behavioral.

## Figures and Tables

**Table 1:** Corruption personnel node adjacency matrix (partly)).

	Node 1	Node 2	Node 3	…	Node 34
Node 1	0	1	0	…	0
Node 2	1	0	1	…	
Node 3	0	1	0	…	
…	…	…	…	…	
Node 34	0	0	0	…	

**Table 2:** Network centrality index of each corruption node.

Node	Degree centrality		Betweenness centrality	Closeness centrality	
	Out-degree	In-degree		Out-degree	In-degree
1	7	1	98	12.222	4.797
2	3	4	192.667	12.268	4.889
3	7	1	74.667	12.268	4.797
4	3	3	5	11.419	4.896
5	6	8	251.833	11.913	4.955
…	…	…	…	…	…
33	1	1	13	10.092	4.867
34	1	1	26	10.927	4.783

**Table 3:** Analysis results of core-edge structure.

Location	Core	Marginal
Personnel number	Node 1, 2, 3, 4, 5, 22, 24	Node 6, 7, 8, 9, 10, 11 et al.

**Table 4:** Probability of each parameter.

Parameters	*λ*	*u*	*r*	*t*
Range	0.239	0.5	0.147	10 years

**Table 5:** Parameter variation relationship of nodes at different crackdown rates.

*γ*	*t*	*S* (*t*)	*t*	*E* (*t*)	*t*	*I* (*t*)	*t*	*R* (*t*)
0.05	70	2	25	3	35	14	120	32
0.1	90	5	30	2	38	6	100	29
0.147	100	12	30	1	40	3	110	22
0.2	120	21	—	—	0	1	120	13

**Table 6:** Parameter variation relationship of nodes with different infection rates.

*λ*	*t*	*S* (*t*)	*t*	*E* (*t*)	*t*	*I* (*t*)	*t*	*R* (*t*)
0.139	100	28	—	—	—	—	110	6
0.239	90	12	30	1	40	3	110	22
0.339	60	6	22	3	30	6	80	28
0.439	50	2	18	4	20	9	60	32

**Table 7:** Parameter variation relationship of nodes with different infection rates.

*t*	*t*	*S* (*t*)	*t*	*E* (*t*)	*t*	*I* (*t*)	*t*	*R* (*t*)
72	—	—	30	1	40	3	—	—
96	—	—	30	1	40	3	—	—
120	90	12	30	1	40	3	110	22
144	90	12	30	1	40	3	110	22

## Data Availability

The data used to support the findings of this study are available from the corresponding author upon request.

## Supplementary Materials

Supplementary Materials The adjacency relationship matrix data used to construct the complex network of Figure 2 will be provided in supplementary materials Annex 1. The complete data of the three centralities used to analyse complex networks in Table 1 will be provided in supplementary materials Annex 2. The original judgment documents used by the literature research room will be provided in supplementary materials Annex 3. The supplementary material Annex 4 contains the source code of the manuscript.
