# Supplementary material for: Characterization of Group Behavior of Corruption in Construction Projects Based on Contagion Mechanism
Source: Comput Intell Neurosci. 2022 Mar 19;2022:8456197. doi: 10.1155/2022/8456197 (PMC8957412; doi:10.1155/2022/8456197)
Supplement: Supplementary Materials — The adjacency relationship matrix data used to construct the complex network of Figure 2 will be provided in supplementary materials Annex 1. The complete data of the three centralities used to analyse complex networks in Table 1 will be provided in supplementary materials Annex 2. The original judgment documents used by the literature research room will be provided in supplementary materials Annex 3. The supplementary material Annex 4 contains the source code of the manuscript. [file 8456197.f1.zip › 8456197.f1/Annex 1 complete data of the three centralities.pdf]

	Closeness centrality			
	inFarness	outFarness	inCloseness	outCloseness
Node1	688	270	4.797	12.222
Node2	675	269	4.889	12.268
Node3	688	269	4.797	12.268
Node4	674	289	4.896	11.419
Node5	666	277	4.955	11.913
Node6	669	1122	4.933	2.941
Node7	669	1122	4.933	2.941
Node8	669	1026	4.933	3.216
Node9	651	1057	5.069	3.122
Node10	1122	1057	2.941	3.122
Node11	601	1089	5.491	3.03
Node12	553	1122	5.967	2.941
Node13	1122	1089	2.941	3.03
Node14	613	1122	5.383	2.941
Node15	1122	263	2.941	12.548
Node16	669	993	4.933	3.323
Node17	669	1122	4.933	2.941
Node18	669	1122	4.933	2.941
Node19	669	1122	4.933	2.941
Node20	647	1122	5.1	2.941
Node21	686	310	4.81	10.645
Node22	683	285	4.832	11.579
Node23	1122	259	2.941	12.741
Node24	620	1023	5.323	3.226
Node25	603	1122	5.473	2.941
Node26	603	1122	5.473	2.941
Node27	603	1122	5.473	2.941
Node28	678	310	4.867	10.645
Node29	698	312	4.728	10.577
Node30	664	1089	4.97	3.03
Node31	1089	238	3.03	13.866
Node32	1089	266	3.03	12.406
Node33	678	327	4.867	10.092
Node34	690	302	4.783	10.927

	Betweenness centrality	
	Betweenness	nBetweenness
Node1	98	9.28
Node2	192.667	18.245
Node3	74.667	7.071
Node4	55	5.208
Node5	251.833	23.848
Node6	0	0
Node7	0	0
Node8	45	4.261
Node9	32	3.03
Node10	0	0
Node11	18	1.705
Node12	0	0
Node13	0	0
Node14	0	0
Node15	0	0
Node16	0	0
Node17	0	0
Node18	0	0

Node19	0	0
Node20	0	0
Node21	0	0
Node22	85.333	8.081
Node23	0	0
Node24	48	4.545
Node25	0	0
Node26	0	0
Node27	0	0
Node28	16.333	1.547
Node29	1.667	0.158
Node30	1.5	0.142
Node31	28	2.652
Node32	0	0
Node33	13	1.231
Node34	26	2.462

		Degree centrality		
	OutDegree	InDegree	NrmOutDeg	NrmInDeg
Node1	7	1	21.212	3.03
Node2	3	4	9.091	12.121
Node3	7	1	21.212	3.03
Node4	3	3	9.091	9.091
Node5	6	8	18.182	24.242
Node6	0	1	0	3.03
Node7	0	1	0	3.03
Node8	1	1	3.03	3.03
Node9	1	1	3.03	3.03
Node10	1	0	3.03	0
Node11	1	2	3.03	6.061
Node12	0	2	0	6.061
Node13	1	0	3.03	0
Node14	0	3	0	9.091
Node15	1	0	3.03	0
Node16	1	1	3.03	3.03
Node17	0	1	0	3.03
Node18	0	1	0	3.03
Node19	0	1	0	3.03
Node20	0	1	0	3.03
Node21	1	1	3.03	3.03
Node22	4	3	12.121	9.091
Node23	2	0	6.061	0
Node24	3	4	9.091	12.121
Node25	0	1	0	3.03
Node26	0	1	0	3.03
Node27	0	1	0	3.03
Node28	1	1	3.03	3.03
Node29	1	1	3.03	3.03
Node30	1	1	3.03	3.03
Node31	2	1	6.061	3.03
Node32	1	1	3.03	3.03
Node33	1	1	3.03	3.03
Node34	1	1	3.03	3.03
